# The planet on our plates: approaches to incorporate environmental sustainability within food-based dietary guidelines

**DOI:** 10.3389/fnut.2024.1223814

**Published:** 2024-07-05

**Authors:** Corné van Dooren, Brent Loken, Tim Lang, Helle Margrete Meltzer, Sarah Halevy, Loes Neven, Kristof Rubens, Marije Seves-Santman, Ellen Trolle

**Affiliations:** ^1^WWF-NL, Zeist, Netherlands; ^2^Wageningen University and Research, Wageningen, Netherlands; ^3^WWF Global Science, Washington, DC, United States; ^4^Centre for Food Policy, City University of London, London, United Kingdom; ^5^Norwegian Institute of Public Health, Oslo, Norway; ^6^WWF-UK, Woking, United Kingdom; ^7^Flanders Institute for Healthy Living (Gezond Leven vzw), Brussels, Belgium; ^8^Department of Environment and Spatial Development (Flemish Government), Brussels, Belgium; ^9^The Netherlands Nutrition Centre(Voedingscentrum), The Hague, Netherlands; ^10^National Food Institute, Technical University of Denmark (DTU), Kongens Lyngby, Denmark

**Keywords:** environmental sustainability, food-based dietary guidelines, food systems, food system transformation, sustainable diets

## Abstract

For many decades, food-based dietary guidelines (FBDGs) were only health-oriented. This changed post-2009 when gradually, an increasing number of countries began to include environmental sustainability considerations in their guidelines. International organisations such as the Food and Agriculture Organization (FAO) and the World Health Organization (WHO) have stated that governments should include environmental sustainability in future FBDGs. However, methodologies on how this should be done are lacking. Therefore, through workshops and discussions with experts, we analysed a selection of methodologies and classified them into six groups: (1) health first; (2) additional advice; (3) demonstrating synergies; (4) modelling impact; (5) combining strategies; (6) systems first. We then assessed how innovative each approach was and their potential for transformative impact. Of the 6 approaches investigated, only approaches 5 and 6 could be considered as disruptive innovations and leading to major changes. Adding environmental sustainability into FBDGs is a policy innovation and has become a debate between old and new multi-criteria guidelines for eating. With the addition of environmental sustainability in FBDGS, a new or emerging set of multi-criteria guidelines for judging food are being proposed that challenges past norms and governance. Today, there is growing scientific consensus that diets that are good for human health are also good for the environment. There is also a growing recognition that food system change is inevitable and desirable. We see this as a positive opportunity to collaborate on FBDGs that are more appropriate for the 21st century and ambitious enough to meet the environmental challenges at hand.

## 1 Introduction

In 1986, Joan Dye Gussow and Katherine Clancy published the first call for dietary guidelines for sustainability (1), and, in so doing, introduced the concept of sustainable diets. Prior to 2010, neither national nor international official guidelines addressed the growing societal concerns around environmental sustainability. In 2010, the FAO and Biodiversity International held an international scientific meeting to address the issue (2). Sweden and Germany were early leaders in developing the concept of healthy and sustainable diets with the Swedish publication of “Environmentally Effective Food Choices” (3) and the German “Sustainable Shopping Basket” (4). Since then, growing evidence about the climate and nature crises have led to a change in policy concern about the relationship between food consumption and the environment, suggesting that food system policies should not only consider the nutritional requirements of present and future generations, but also how food is produced, processed and consumed. This is especially urgent given that food systems are the leading cause of environmental harm and degradation of natural ecosystems in ways that threaten economic, social, and environmental viability (5).

Since 2010, the FAO has recommended the development of FBDGs that promote healthy diets but also take into consideration their impact on global and local agri-food systems while being culturally and socio-economically appropriate (2). In 2019, the FAO and WHO organised an expert meeting to describe sustainable healthy diets. The conclusion was that:

Sustainable healthy diets are dietary patterns that promote all dimensions of individuals’ health and wellbeing; have low environmental pressure and impact; are accessible, affordable, safe, and equitable; and are culturally acceptable. The aims of sustainable healthy diets are to achieve optimal growth and development of all individuals and support functioning and physical, mental, and social wellbeing at all life stages for present and future generations; contribute to preventing all forms of malnutrition; reduce the risk of diet-related NCDs; and support the preservation of biodiversity and planetary health. Sustainable healthy diets must combine all the dimensions of sustainability to avoid unintended consequences (6).

At the same time, the FAO and WHO formulated Sustainable Healthy Diets Guiding Principles. These put forward eight principles regarding health, five regarding environmental sustainability, and three regarding sociocultural aspects. The principles of environmental sustainability are to:

- Maintain greenhouse gas (GHG) emissions, water and land use, nitrogen and phosphorus application and chemical pollution within set targets
- Preserve biodiversity, including crops, livestock, forest-derived foods, and aquatic genetic resources, and avoid overfishing and overhunting
- Minimise the use of antibiotics and hormones in food production
- Minimise the use of plastics and derivatives in food packaging
- Reduce food loss and waste (6).

In addition, a task force of the Federation of European Nutrition Societies (FENS) took the position that future dietary guidelines should include environmental sustainability and that countries require guidance on how to best integrate the different dimensions of environmental sustainability in ways that reflect both their national priorities and global commitments. This FENS task force concluded that further work was needed to explore current practices, existing methodologies, and future prospects for incorporating other relevant dimensions within a future conceptual framework for sustainable food-based dietary guidelines (FBDGs) in Europe (7). The present study contributes towards the creation of such a framework.

Independently of these efforts, the EAT-Lancet Commission on Healthy Diets from Sustainable Food Systems (8) developed two sets of scientific targets, one for healthy diets and another for sustainable food production within planetary boundaries (PBs). The EAT-Lancet Commission then modelled whether it was possible to achieve healthy diets from sustainable food systems for a global population of 10 billion people by 2050 and determined that it was possible but only with radical changes to how we produce our food, what we eat, and how much food is lost or wasted. The commission put forth five strategies of achieving healthy diets from sustainable food systems, the first of which was to seek international and national commitments to shift toward healthy diets (8, 9).

Numerous studies have concluded that existing FBDGs not only improve health but are generally more environmentally sustainable than the typical Western diet (10–13), i.e., there is a substantial overlap between public health and environmental sustainability goals. The correlation between healthier foods and lower environmental impact is in line with the findings of the EAT-Lancet Commission, which showed strong synergies between an optimal healthy diet and lower environmental impact. Clark et al. (14) also found that foods associated with the largest negative environmental impact – that is, unprocessed and processed red meat – are consistently associated with the largest increases in disease risk. Thus, dietary transitions towards greater consumption of healthier foods would improve environmental sustainability, although processed foods that are high in sugar harm health but can have a low environmental impact (14).

Nevertheless, while some of the current FBDGs, if implemented, are associated with moderate improvements in health and reductions in environmental impacts at the national level, most FBDGs are incompatible with achieving health and environmental targets. Springmann et al. (15) concluded that most FBDGs they examined were not compatible with a set of global environmental targets including the 2015 Paris agreement to limit global warming to below 2°C, the Aichi biodiversity target of limiting the rate of land use change, the Sustainable Development Goals (SDGs) and planetary boundaries related to freshwater use, and nitrogen and phosphorus pollution. Loken et al. (16) came to a similar conclusion and found that current FBDGs of the G20 countries will not ensure that global warming remains below 1.5°C and should be further improved to ensure they are more ambitious and in line with global health and environmental targets.

Although previous studies focused on the content of FBDGs in relation to environmental sustainability – the “what” (17, 18) – this paper is the first to focus on the approaches used by countries to incorporate environmental sustainability in FBDGs – the “how.” This is important because to date, there is no coherent, integrated, unified approach to developing FBDGs that are in line with the latest science on healthy diets from sustainable food systems (19).

## 2 Methods

This study is based on a literature review of publications and reports about FBDGs in relation to environmental sustainability that were published between 2009 and 2022, along with input, analysis and conclusions derived from workshops and discussions with experts. As of 2022, FAO has identified 95 countries with FBDGs (20). A recent review of FBDGs around the world assessed 83 countries and found that 37 referred to environmental sustainability (18). Out of the 37 countries that referred to environmental sustainability in their FBDGs, this study selected examples whose methods and approaches were published in English and who were transparent about both the methodology and approach used and how the guidelines were developed.

The FBDGs of these countries were assessed with respect to how they analysed, described and addressed the priorities of human health and environmental sustainability (20–22). The authors assessed these approaches during a series of workshops that were organised to help develop new FBDGs. These included an FAO organised workshop at Wageningen University and Research (23), a follow-up workshop in Rome with the Technical and Advisory Group for Updating the Global Guidance for the Development/Revision of FBDGs (2019) and a WHO hosted meeting where experts presented the latest evidence on new approaches on FBDGs (24). In addition, authors presented and participated in workshops of Deutsche Gesellschaft für Ernährung in Bonn (25), the European Public Health Nutrition Alliance (online meeting EPHNA, 2020), the Flanders Institute for Healthy Living in Brussels (2021), the Health Council of the Netherlands in The Hague (2023) and the Nordic Nutrition Recommendations in Oslo and online [webinar; (26)].

Our paper provides an overview of the approaches used to date to address health and environmental sustainability in dietary advice at the national level and the impact each strategy could have in achieving health and environmental goals. The six approaches outlined here are:

1. Health first
2. Additional advice
3. Demonstrating synergies
4. Modelling impact
5. Combining strategies
6. Systems first.

Table 1 outlines these six approaches and illustrative examples from countries around the world are used to demonstrate how they have been used to develop FBDGs.

**Table 1:** The six approaches used by countries to develop FBDGs and the level of impact that each approach may have in achieving health and environmental sustainability goals.

Approach	Description	Health versus environmental sustainability	Example(s)	Potential level of impact
1	Health first	Health is the focus with no mention of environmental sustainability: traditional FBDGs.	United States Low- and middle-income countries (LMICs) Brazil	Incremental
2	Additional advice	Health is the focus with some additional attention given to the environment.	Nordics United Kingdom (UK)	Incremental
3	Demonstrating synergies	Health is first but synergies with environmental sustainability are also considered as long as they support health goals.	Canada Belgium	Architectural
4	Modelling impact	Modelling is used to demonstrate impact. Health and environmental sustainability are both taken into account.	France Germany	Diversifying
5	Combining strategies	Health and environmental sustainability are taken equally into account and a combination of approaches 1 to 4 are used.	Netherlands Denmark	Diversifying or path-breaking
6	Systems first	Food systems are the focus with health, socio-cultural and environmental sustainability considered as part of a healthy and sustainable food system and a combination of strategies are used.	None	Path-breaking

**Figure 1:**
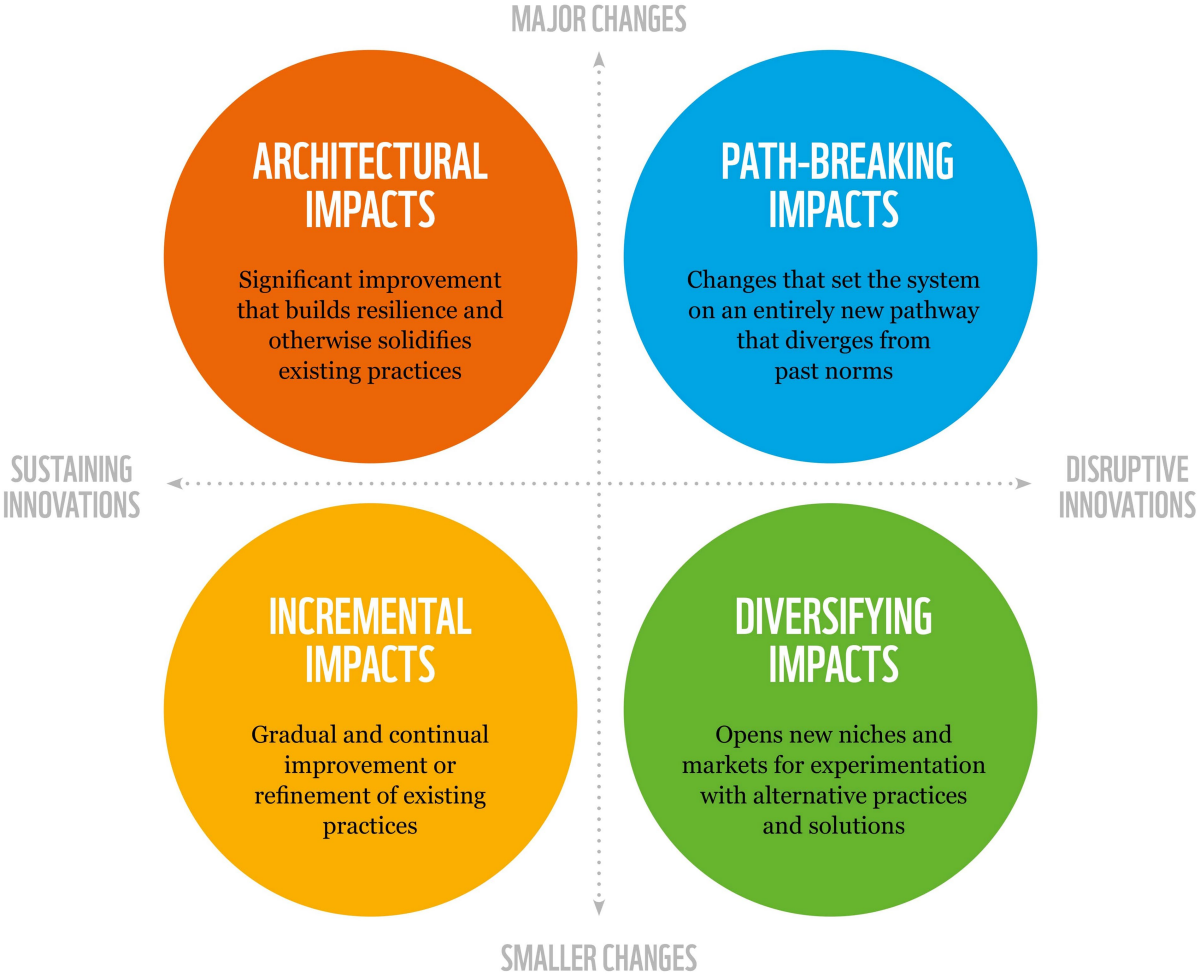
Food based dietary guidelines can be seen as a policy innovation, either sustaining or disrupting existing ways of doing things and creating space for something new to emerge. The impacts on the food system of these policy innovations can be smaller and introduce new ideas or approaches to existing practices, or they can be major, representing investments in the architecture and infrastructure of existing systems or completely reorienting people’s practices, habits, and goals [adapted from (28) with permission].

The development of FBDGs that incorporate environmental sustainability can be seen as a policy innovation. Innovations can be categorised depending on changes they provide. Innovations may be disruptive when they destabilise existing norms, institutions and markets (27). They may shake up existing systems through diversifying impacts, which open new niches and markets, or they can create path-breaking impacts, driving the emergence of entirely new practices that previously would have seemed unfeasible (28). Innovations can also be sustaining when they support existing norms, institutions and markets. Four categories or types of impact are indicated as possible: architectural, path-breaking, incremental and diversifying (Figure 1). For this paper, we used an innovation lens to assess the various approaches used to develop FBDGs and then used this lens to assess the potential level of impact each approach may have toward shifting diets to help achieve health and environmental goals. However, the actual impact depends on the additional initiatives and policies put in place to enable implementation, i.e., FBDGs alone are not enough to transform consumption patterns. To make real systemic transformation happen, the establishment of not just an enabling but a normative environment, guiding the innovation process is needed (29).

It’s important to note that the desired level of impact depends on the starting place of the system itself and where science indicates where the system should be. If, for example, the starting point of a country’s FBDGs are guidelines that focus only on health, then the desired state would be toward the development of more disruptive FBDGs that consider health and environmental sustainability equally, as supported by science. However, if the starting point are FBDGs that already consider health and environmental sustainability equally, then future FBDGs would require a sustaining policy innovation (i.e., incremental or architectural impact). In addition, whether a country requires or can tolerate smaller or major changes needs to be carefully considered by policy makers. Sometimes, smaller changes (i.e., diversifying impact) may receive broader acceptance from the public than major changes (i.e., path-breaking impact).

## 3 Results

Six approaches used by countries to incorporate environmental sustainability into FBDGs were assessed (Table 1). Each approach can be distinguished from others, based on whether the FBDGs can be seen as either a sustaining or disruptive policy innovation and also the level of impact (i.e., smaller or larger) each approach may have toward shifting diets to help achieve health and environmental goals.

### 3.1 Approach 1: health first

The Health First approach distinguishes itself by focusing mainly on health in FBDGs. Most of the 95 countries that have FBDGs apply this traditional, health focussed approach (20). The methodology used to provide health advice in FBDGs, based on the latest science, has developed significantly over the last couple of decades with the goal of being transparent and reducing bias (30). According to the definition from the FAO, Health First dietary guidelines are intended to establish a basis for public food and nutrition, health and agricultural policies and nutrition education programmes to foster healthy eating habits and lifestyles. They provide advice on food, food groups and dietary patterns with the explicit purpose of informing the public about the foods and nutrients that are required to promote overall health and prevent chronic diseases (20). The advantages and disadvantages of this and the other approaches are summarised in Table 2.

**Table 2:** The advantages and disadvantages of the six approaches discussed.

	Approach	Advantages	Disadvantages
1	Health first	A lot of experience and science available to develop traditional FBDGs.	Hardly any attention to environmental sustainability.
2	Additional advice	This is the simplest and easiest approach to apply because it adds new rules to existing FBDGs.	It could limit consumer choice.
		This could be both qualitative and sometimes quantitative advice, depending on the scientific evidence available.	It could conflict with health advice and nutritional requirements.
		It builds on scientific evidence and experiences from one’s own or other countries.	Consumers might see it as adding complexity or being of less importance than the health advice.
		Cost-effective to construct.	It is based on measures, not on outcomes.
			It requires additional food literacy from consumers by asking them to interpret ‘seasonal,’ ‘local,’ ‘sustainable’ and other labels.
3	Demonstrating synergies	Provides a more integrated message covering health and environmental impacts.	No attention is paid to conflicts between health and environmental sustainability, such as advice on sugars.
		Supports traditional diets, such as the Mediterranean and Nordic diet, and is adaptive to native cultures.	Difficult to manage trade-offs between environmental indicators or satisfy several constraints simultaneously.
		Quantifying the synergy can help consumers make more informed choices.	This misses additional environmental gains or a food system approach.
		Growing evidence from science and practice.	Mostly focused on nutritional health but not on other health issues such as zoonoses and antimicrobial resistance.
4	Modelling impact	It provides optimised solutions while satisfying several constraints, such as health, environmental sustainability, and costs.	This needs the use of sophisticated tools, experts, and data can be expensive.
		Contrary to scenarios, optimisation tools can calculate solutions that are close to the current diet.	Interpretation of outcomes requires dedicated experts, without guaranteeing satisfying, realistic solutions.
		Less conflicts between health and environmental advice.	This requires significant (national) environmental and consumption data, which are not available for all essential food products or all environmental aspects. Thus, it may be difficult to apply in low to middle income countries.
			Introducing acceptability constraints is recommended, however no study has as yet provided an ultimate solution for calculating acceptability.
5	Combining strategies	This approach could indicate which products are best to consume more frequently or less frequently if you want to eat healthier and more sustainably.	There is a lack of good practices applying optimisation or other multi-criteria techniques to FBDGs.
		This approach is more focused on the metrics of complete diets.	Introducing acceptability constraints is recommended, however no study has as yet provided an ultimate solution for calculating acceptability.
6	Systems first	A food system approach can help prioritise competing demands.	This needs the use of sophisticated tools, experts, and data can be expensive.
		Guarantees achieving multiple planetary boundaries and some SDGs at the same time.	The methods and applications are still under development and need much expertise on indicators and planetary boundaries.
		If well applied, this could also support other sustainability dimensions such as social-economic and animal welfare.	This requires significant (national) environmental and consumption data, which are often not available for all essential food products. Thus, it is difficult to apply to low to middle income countries.
			This approach needs a lot of expertise on environmental indicators and setting ambitious targets.

#### 3.1.1 United States (2015, 2020)

In 2015, the US Dietary Guidelines Advisory Committee (DGAC) tried to incorporate environmental sustainability when developing their guidelines. The DGAC concluded: “Consistent evidence indicates that, in general, a dietary pattern that is higher in plant-based foods, such as vegetables, fruits, whole grains, legumes, nuts, and seeds, and lower in animal-based foods, is more health promoting and is associated with lower environmental impact (e.g., GHG emissions, land and water use, biodiversity loss) than the current average US diet.” This can be achieved without excluding any food groups (31). The DGAC (31) also concluded that sustainable solutions can be achieved through diverse types of diets, such as vegetarian, Mediterranean or DASH (Dietary Approaches to Stop Hypertension) diets. The DGAC proposals received a hostile reception from the US meat industry. Despite public support, the DGAC’s recommendations to combine nutritional and sustainability advice was not approved in the Senate, and the environmental dimension was dropped entirely (32). The updated 2020–2025 US Dietary Guidelines provides four overarching guidelines that encourage healthy eating patterns at each stage of life and recognise that individuals will need to make shifts in their food and beverage choices to achieve a healthy pattern. However, these updated guidelines still do not take into account environmental sustainability and the only reference to environmental issues is regarding seafood consumption and limiting methylmercury exposure for women who might become pregnant or lactating (33).

#### 3.1.2 Brazil (2014)

The 2014 Brazilian guidelines were produced after public consultation (34). They are distinguished by the fact that they emphasised the social and economic aspects of sustainability, advising people to be wary of, amongst other things, advertisements, and to avoid ultra-processed foods that are not only bad for one’s health but also undermine traditional food cultures. This focus contrasts with the largely environmental definition of sustainability used in other guidelines (17). Nevertheless, like other countries, Brazil advised consumers to base their diets on many varieties of natural or minimally processed foods, mainly of plant origin. Vegetables and fruits that are local or in season are presented as the best choices, as are fruits produced agro-ecologically. Consumers are advised to limit the use of processed foods, consuming them only in small amounts as ingredients in culinary preparation or as part of meals based on natural or minimally processed foods. By advising more traditional recipes, the reduction of animal-based products is indirectly advised: ‘In most traditional cuisines, meat, fish, eggs and other animal foods are consumed sparingly, as part of dishes and meals that are based on plant foods,’ and ‘Small changes in the diets of the Brazilians who eat most natural or minimally processed foods – such as eating more vegetables and less red meat – would render the nutritional profile of their overall diet practically ideal.’ They call it the Golden Rule: “Always prefer natural or minimally processed foods and freshly made dishes and meals to ultra-processed foods,” without referring to the environment directly (34).

#### 3.1.3 Low- and middle-income countries

Until now, the majority of lower-middle-income countries with FBDGs use a health first approach for their FBDGs. To date, only a handful of countries have references to environmental sustainability (e.g., Ethiopia, Colombia, Ecuador, El Salvador), compared to 34 upper-middle/high-income countries (18). The FAO is assisting member countries in developing, revising, and implementing their FBDGs and food guides, in line with current scientific evidence. To assist this process, the WHO is developing a user-friendly open access data platform which enables Member States to model diets using their own national datasets. These models can then be used to adopt local diets in order to meet health and sustainability goals (24).

#### 3.1.4 Level of impact

The Health First approach can be considered as having an incremental impact (sustaining innovation and smaller changes - Figure 1) since they sustain the status quo of focusing mainly on health, thus leading to smaller changes in current dietary patterns and people’s practices, habits, and goals. Although this approach may improve existing FBDGs, countries that use this approach are still failing to incorporate the advice of intergovernmental bodies such as the FAO and WHO on the need to incorporate environmental sustainability. In addition, this approach fails to take into account the overwhelming scientific evidence on the relationship between health and the environment, therefore sustaining the status quo when it comes to dietary shifts and not creating systemic changes to support healthy and sustainable diets. However, the level of impact of a policy can change over time. Brazil’s FBDGs could be considered as a disruptive policy innovation when they were developed, but they have now been largely surpassed by many other countries and the scientific evidence on health and the environment has evolved. The 2020 US FBDGs are also incremental in that they again focused only on health and ignored environmental sustainability. Lastly, although some LMICs have started to address environmental sustainability, more support will be needed in order to have more path-breaking impact.

### 3.2 Approach 2: additional advice

This approach begins with traditional advice on a healthy diet and then supports this with additional advice on environmental sustainability. This approach is generally based on a literature review of the environmental impacts of different foods and diets. It aims to limit the environmental impact of diets in line with existing health based FBDGs through advice on maximum or minimum consumption quantities, choice editing, and food group specifications. Countries that have applied this approach are, for example, Finland (2014), Sweden (2015), Estonia (2015), United Kingdom (2016), and Spain (2018). Frequently used messages are to eat less animal-based and more plant-based foods, choose certified seafood, drink tap water, eat local and seasonal fruits and vegetables, and waste less food (17, 18, 35). This advice is mostly qualitative and has the potential to support achieving some of the Sustainable Development Goals (SDGs) (36, 37) and is in line with the five guiding principles of FAO/WHO, mentioned in the introduction (6). The Additional Advice approach is the simplest and easiest approach to integrate environmental sustainability because it adds new guidance to already existing FBDGs, but could also limit consumers choice, by adding more complexity and requiring additional food literacy. The advantages and disadvantages of this and the other approaches are summarised in Table 2.

#### 3.2.1 Nordics (2012, 2023)

The application of additional advice on environmental sustainability began in the Nordics with the 2012 Nordic Nutrition Recommendations (NNR2012) (38) which was mainly based on initiatives in Sweden (3, 39) and in Finland (40). In general, the NNR advised that a more sustainable diet consisted of more plant-based foods and less animal-based food (e.g., meat, dairy, and eggs). With a goal of reducing GHG emissions, the report put forward five guiding principles for consumers:

1. Choose primarily meat and fish with low environmental impact
2. Eat more dried beans, peas, lentils, and cereals
3. Mainly choose field vegetables, root vegetables, potatoes, fruits, and berries that store well
4. Choose perishable products when they are in season; and.
5. Minimise waste (38).

The purpose of the NNRs is to provide the five Nordic countries (and three Baltic countries added in 2023) with updated scientific advice as a basis for later country specific FBDGs. The Nordics have developed common evidence-based recommendations on nutrient intake since 1980. This is a unique international collaboration that underscores the importance of cooperation between countries, combining their efforts and sharing costs. Their methodological approach has been described in detail in Christensen et al. (41).

Sweden was the first Nordic country to integrate these guiding principles into their FBDGs. Sweden’s official 2015 guidelines were presented as ‘find your way to eat greener, not too much, and be active’ and provided guidance on how to eat healthily and in an environmentally friendly manner (39). The main aim was to encourage consumers to eat less meat and meat products and more plant-foods, including whole grains, vegetables and fruit, as well as healthy oils and some fish to decrease the risk of common chronic diseases in Sweden. In Finland, although additional advice on environmental sustainability was not fully included in the Finnish 2014 FBDGs (40), they did advise ‘Weight control for reasons of sustainability’. Recent advice from the Finnish Food Authority does provide additional advice on environmental sustainability, including that food should be considered in the broader context of the environment and sustainable development (40).

In 2023, the Nordic Nutrition Recommendations (NNR2023) were updated and included environmental sustainability based on the latest scientific evidence (30). The parameters related to environmental sustainability were GHG emissions, land and water use, biodiversity loss, phosphorus flows and industrial chemicals. The NNR2023 provides a framework for the national authorities to develop FBDGs in the Nordic and Baltic countries. NNR2023 recommends a predominantly plant-based diet high in vegetables, fruits, berries, pulses, potatoes and whole grains. It also recommends an ample intake of nuts and fish from sustainably managed stocks, moderate intake of low-fat dairy products, and limited intake of red meat and poultry. The scientific advice on food intake for each food group was established in a transparent way and first considered the evidence on human health. If the scientific evidence was strong, quantitative health-based advice was given, otherwise qualitative advice was given. Environmental impact was considered next and only if advice on reducing the environmental impact could narrow the health defined intake ranges without compromising health. If this was possible, only then the environmental impact influenced the science advice given (30). The NNR2012 guidelines are considered as Approach 2, while the more comprehensive guidelines in NNR2023 are considered as a combination of Approaches 2 and 3. The Nordic and Baltic countries are in the process of implementing the NNR2023. For example, Norway has revised their FBDGs, which were sent out for public consultation in April 2024 (42).

#### 3.2.2 United Kingdom (2016)

The British Eatwell Guide (43) took an initial step towards environmental sustainability by recommending: ‘Eat more beans and pulses and consume two portions of sustainably sourced fish a week, one of which is oily. Eat less red and processed meat.’ The names of the food group segments have been revised to emphasise certain foods within the food group that are deemed to be more environmentally friendly, such as, for example, the protein segment being called ‘Beans, pulses, fish, eggs, meat, and other proteins.’ This serves to demonstrate that the nutritional value of proteins from plant sources is an important part of one’s total protein intake. The daily recommendation of protein was also reduced from 15% of total energy to 8%. When consumers follow these guidelines, it is estimated to have a significant reduction in GHG emissions. This reduction is due to several changes in food choices, such as increased consumption of potatoes, fish, wholemeal bread, vegetables and fruit, while simultaneously reducing the amounts of dairy, meat, rice, pasta, pizza and sweet foods (44). Drinks were not included in the study. The British Eatwell Guide (2016) also used linear programming (LP - see Approach 4) to optimise nutrition and affordability but did not include the environment (45, 46).

#### 3.2.3 Level of impact

The Additional Advice approach can be considered as having an incremental impact (sustaining innovation and smaller changes - Figure 1) since they mainly sustain the status quo of focusing predominantly on health, thus leading to potentially smaller changes in current dietary patterns and people’s practices, habits, and goals. Although adding some advice on environmental sustainability is a positive step, this approach still fails to go far enough and fully integrate environmental sustainability as recommended by FAO, WHO and the latest scientific evidence of what is needed to achieve both health and environmental goals, thus mainly sustaining the status quo when it comes to dietary shifts and not creating systemic changes to support healthy and sustainable diets. The Nordic countries in particular, with the exception of Denmark, have so far made only incremental steps since being a front-runner in aiming to integrate environmental sustainability in FBDGs. Although this approach does support achieving some of the SDGs, it is not expected to result in major changes or quantified, path-breaking impacts on achieving environmental sustainability goals.

### 3.3 Approach 3: demonstrating synergies

The Demonstrating Synergies approach explores the synergies between health and environmental sustainability on integrated messages related to health and the environment. This approach also considers current and historical dietary perspectives, supports traditional diets and is adaptive to native cultures. Health is still first with this approach, but environmental sustainability is also largely considered as long as it supports health goals. This approach highlights, for example, that the reduction of the consumption of red and processed meat is both good for your health and the environment. Examples of applying this approach are Canada (2019) and Flanders, Belgium (2017; 2021).

Research supports the use of this approach for achieving both health and environmental goals. For example, the traditional Mediterranean diet (10, 47), the New Nordic Diet (48) and the traditional diet of the Netherlands and Belgium (49) all have the potential for achieving health goals while reducing environmental impacts. This synergy can be explained by a common feature of these diets: they have high nutrient density and low energy density [i.e., high in proteins, vitamins and minerals and low in calories per gram (50)]. Quantifying the degree of synergy found through a simple and consistent method can help consumers make informed choices (51). Such a method can indicate which foods consumers should eat more or less of if they want to live a healthier and more sustainable life. The advantages and disadvantages of this approach are summarised in Table 2.

#### 3.3.1 Canada (2019)

While health is the primary focus of Canada’s FBDGs (52), the potential environmental benefits associated with shifting diets is also outlined. For example, the guidelines highlight that there is scientific evidence to support that eating more plant-based foods and less animal-based foods has a smaller environmental impact. In addition, the guidelines outline that reducing food waste by households, food manufacturers and processors, farmers and food retailers can significantly reduce the environmental impact of food systems. Attention is also paid to eating with others and the social, cultural and historical context of Indigenous peoples: ‘Indigenous knowledge is key for sustainable harvesting and cultivation, as well as for the preparation, storage, consumption, and sharing of traditional food’ (52).

Canada’s FBDGs recommends general protein-rich foods, with a preference for plant-based foods (in the following order): ‘Protein foods: include legumes, nuts, seeds, tofu, fortified soy beverages, fish, shellfish, eggs, poultry, lean red meats including wild game, lower fat milk, lower fat yoghurts, lower fat kefir, and cheeses lower in fat and sodium.’ The guidelines also emphasise water as the preferred drink. Canada’s FBDGs (52) made headlines due to the belief that dairy would no longer be recommended, but in fact dairy remains a part of the recommended protein-rich food group. In addition, the guidelines advise limiting highly processed foods (52).

#### 3.3.2 Belgium (2017; 2021)

In 2017, the Flemish food triangle was thoroughly revised, based on the latest scientific evidence within the fields of nutrition, health, and behavioural sciences (53) and updated with new evidence in 2021 (54). The Flemish Institute of Healthy Living (“Vlaams Instituut Gezond Leven”) provided food guidelines that should be achievable in the long term for the general population, and which should be easily accessible and convenient to understand in what is called ‘the Food Triangle’. This triangle classifies food products according to their health and environmental impact, using colour coding for clarity, similar to the Barilla double pyramid (10) and Sustainable Nutrient Rich Foods-index (55). The result is a set of seven practical recommendations which integrate health and environmental sustainability and are based on three main principles:

1. Eating more vegetable than animal foods
2. Avoiding ultra-processed foods (empty calories) as much as possible by focusing more on fruit, vegetables, whole grains, legumes and nuts; and
3. Not wasting foods and moderating overall consumption (53).

The environmental impact of food was considered for the first time in the 2017 revision of the Food Triangle, however their inclusion did not follow the same rigorous scientific process as for health. Consequently, these guidelines lacked a robust foundation (54) and the environmental claims of these guidelines were called into question by the agriculture and food sectors. To address these concerns, the Flemish Institute for Healthy Living, the Agency for Health and Care, and the Department of Environment and Spatial Development collaborated in 2021 to better substantiate and fully integrate environmental sustainability in their guidelines. They did this by publishing a new background document which followed the same rigorous scientific process which was used for health in 2017 (54). The conclusion was that, in most cases, environmental sustainability and good health go hand in hand when it comes to food recommendations.

#### 3.3.3 Level of impact

The Demonstrating Synergies approach can be considered as having an architectural impact (sustaining innovation and larger changes - Figure 1) since they still mainly focus on health. However, since synergies with environmental sustainability are also considered, this could potentially lead to major changes in current dietary patterns and people’s practices, habits, and goals. Although this approach does make improvements on previous versions of FBDGs and has the potential for major changes in consumption patterns, this approach may still sustain many existing food system practices that could hinder their adoption. This approach has a lot of potential to reduce environmental impacts, but in itself the guidelines do not promote changes in the food system and may not necessarily lead to a more systemic change. However, adoption of the dietary shifts put forth by this approach may be more likely than approach 2, because it integrates messages for both human health and environmental sustainability, is more culturally acceptable by building on traditional diets and indigenous food cultures, and helps consumers make more informed choices.

### 3.4 Approach 4: modelling impact

The Modelling Impact approach is dietary modelling with the intent of reducing environmental impacts. Health and sustainability are taken equally into account by using constraints on each. Linear programming (LP) is often applied to find an optimal diet while also satisfying several constraints (i.e., health, affordability and environmental sustainability) at the same time (56, 57). In (2011), the World Wide Fund for Nature UK used LP to propose the Livewell Plate, which was a variation of the official UK Eatwell Plate (58, 59). WWF updated this in 2023, again using optimisation modelling, and demonstrated enormous potential for reducing GHG emissions while providing healthy and affordable diets for UK citizens (60). Only the Netherlands (2016), France (2016), and Germany (2024) have applied LP in their official FBDGs using environmental constraints. LP exhibits potential as an instrument for finding solutions to a variety of complex dietary problems. Future possibilities include finding solutions for an optimal diet by combining nutrition, cost, environmental and acceptability constraints (57). The advantages and disadvantages of this approach are summarised in Table 2.

#### 3.4.1 France (2016)

The French Agency for Food, Environmental and Occupational Health & Safety (ANSES) developed a digital tool to optimise food consumption for the French FBDGs in 2016. This tool calculates combinations of food groups that meet the stated goals in the FBDGs. This includes meeting the nutritional needs of its citizens, preventing chronic non-communicable diseases, and minimising exposure to environmental-related food contaminants (pesticide residues and heavy metals), all while keeping food intake close to current levels of consumption. The French included contaminants in their FBDGs to limit the risk associated with foodborne contaminants, such as pesticide residues, however, other environmental variables were not modelled. In the end, it was difficult to reach all three goals without changes to current consumption patterns that included either lowering the nutritional recommendations for vitamin D intake or increasing the maximum exposure limit to contaminants (61).

#### 3.4.2 Germany (2024)

For the development of the updated German FBDGs, European scientists were invited by DGE (*Deutsche Geselschaft für Ernährung*) and Federation of European Nutrition Societies (FENS) in 2019 to discuss using mathematical optimisation to integrate both health and environmental sustainability into their new FBDGs. The scientists concluded that mathematical optimisation is a suitable tool for finding trade-offs between conflicting goals and considering multiple dimensions in FBDGs and can actually increase consumer acceptance for dietary shifts (25). In cooperation with the French MS Nutrition, the DGE developed the German Nutrition Optimisation Model (GNOM) for its revised FBDGs (62). The LP model considered three variables to find an optimal diet that would minimise environmental impact (GHG emissions and land use), diet-related health burden (disability adjusted life years) and the relative deviation from current dietary intakes (cultural acceptability). Moreover, deviations away from the nutritional needs of 39 nutrients were also minimised, which, in turn, resulted in a recommended increase in consumption of fruits, vegetables and whole grains, and a reduction in red meat. Germany also took into account co-products in the food system, by using a ratio for, e.g., milk (products) vs. butter and beef from dairy cows and a ratio for red meat (unprocessed vs. processed) (62, 63). The new FBDGs were published in 2024, together with a new food circle with the main conclusion being that “a healthy and environmentally friendly diet is more than 3/4 plant-based and almost 1/4 animal-based” [i.e., in grams of products (64)].

#### 3.4.3 Level of impact

The Modelling Impact approach can be considered as having a diversifying impact (disruptive innovation and smaller changes - Figure 1) since they disrupt the status quo by focusing both on health and environmental sustainability and use a new method (i.e., modelling) that opens up new possibilities for exploration of FBDGs. However, by itself this approach may lead to mainly smaller changes in the food system, especially by keeping proposed dietary changes as close as possible to current consumption patterns. However, in some cases smaller changes (i.e., no need to set the system on an entirely new pathway) may be all that are needed and one advantage of the modelling impact approach is that contrary to proposing dietary patterns as a scenario (i.e., vegetarian, vegan, Mediterranean diets), modelling can calculate solutions that are as close to current consumption patterns as possible. Although this may lead to mainly smaller changes in the system, this may lead to higher levels of consumer acceptance by offering concrete, quantified dietary advice on how to achieve both human health and environmental sustainability outcomes while staying as close to current consumption patterns as possible.

### 3.5 Approach 5: combining strategies

The Combining Strategies approach could be thought of as either a separate approach or as different steps within a policy process with the ultimate goal of fully integrating environmental sustainability and public health advice on nutrition and potentially setting the food system on an entirely new path that differs from past norms. Examples are the Netherlands (2016; combining 2, 3 and 4) and Denmark (2021; combining 3 and 4). The advantages and disadvantages of this approach are summarised in Table 2.

#### 3.5.1 The Netherlands (2016)

In 2011, the Health Council of the Netherlands gave additional advice on environmental sustainability (Approach 2) when they concluded that a shift from the current Dutch diet towards the healthy diet described in the dietary guidelines was good not only for health but also for the environment (11). The Health Council of the Netherlands published new dietary guidelines in 2015 with the main recommendation being to eat less animal-based foods and more plant-based foods, specifically to limit the consumption of red and processed meat [Approach 3 - (65)]. These guidelines underpinned the most recent Wheel of Five, which is the educational model used by the Netherlands Nutrition Centre to help consumers make their diets healthier and more environmentally sustainable (66). In the 2016 update of the Wheel of Five, for the first time, a clear quantitative recommendation was provided regarding the maximum amount of meat to be consumed (500 g of total meat and a maximum of 300 g of red meat per week). The advice was to ‘vary your diet with fish, legumes, nuts, eggs and vegetarian products’ - with a lower environmental impact than meat - and to eat a weekly portion of legumes (135 g) and a handful of nuts per day (25 g). In addition, seven practical ways (Approach 2) to achieve a more sustainable diet (66, 67) were given and subsequently updated in 2020.

Modelling impact (Approach 4) was also used in 2016 in the development of the Wheel of Five. In this process, the Dutch dietary guidelines, Dietary Reference Values (DRV), and current Dutch consumption patterns were considered in an optimisation model and combined with expert judgement. Several maximum restrictions were set for food groups due to environmental sustainability and feasibility while staying as close as possible to people’s current consumption patterns, but no restrictions were set on environmental indicators (66). Subsequently, the environmental impact of the Dutch FBDGs was calculated together with the National Institute for Public Health and the Environment. The conclusion was that a diet based on the Wheel of Five, rather than the current diet, resulted in a slightly reduced environmental impact for men (−13% in GHG emissions). However, higher reductions could be achieved by making more sustainable food choices, for example, when meat was replaced by pulses, nuts and eggs (68). This additional advice (Approach 2) was meant to help consumers to further reduce the environmental impact of their diets.

#### 3.5.2 Denmark (2021)

Denmark’s FBDGs (2021) not only advises Danes on how to eat more healthily but also how their diet can be more climate friendly (69). Specifically, the FBDGs advises Danes to eat plant-rich, varied foods, and not to eat too much. The Danish approach clearly sets out to find synergies with health and environmental sustainability (Approach 3) but by using practical advice for consumers. This practical dietary advice appears to be inspired by the much-cited work of the journalist Michael Pollan, who famously wrote: ‘Eat food, not too much, mostly plants’ (70). Also more concrete advice is given such as eat more vegetables and fruits (600 g/day), eat less meat, choose pulses and fish (350 g of meat (including poultry) per week is sufficient; 350 g of fish per week, 200 g being fatty fish; 100 g pulses per day should be consumed; and 30 g of nuts per day and 1–2 spoons of seeds are appropriate). Also, more food groups are recommended such as eating foods with whole grains, choosing vegetable oils and low-fat milk and dairy, and eating less sugar, fat, and salt containing foods. Finally, the guidelines draw attention to reducing food waste (69).

These quantities are largely in line with the dietary recommendations of the EAT-Lancet Commission on Healthy Diets from Sustainable Food systems (8). The Danish plant-rich diet was modelled (Approach 4) in accordance with the EAT-Lancet Commission’s global reference diet but also takes national food availability and culture into account (71). This was done by using Danish food consumption data as a starting point for the modelling, also including processed foods, discretionary foods and beverages. In addition, the modelled intake was adjusted so that it was in line with the latest scientific evidence on the relationship between food intake and disease risk, while also ensuring that it was in accordance with nutrient recommendations [NNR2012; (72)]. Further, the climate impact of the Danish plant-rich diet was estimated (73).

#### 3.5.3 Level of impact

The Combining Strategies approach can be considered as having an impact between diversifying and path-breaking (disruptive innovation, smaller to major changes - Figure 1). These FBDGs as a policy innovation can be considered disruptive, given that health and environmental sustainability are taken equally into account, however the level of impact could be anywhere from smaller to major depending on the level of dietary shifts recommended or systemic changes required. The Netherlands FBDGs modelled dietary shifts which would be as close as possible to people’s current consumption patterns (i.e., smaller changes), whereas Denmark recommended larger dietary shifts (i.e., larger changes) in line with the EAT-Lancet Commission. However, setting stricter environmental constraints may also shape optimised diets that deviate more from the current consumption and would have more of a path-breaking impact. In addition, the combined strategy has the potential to be path-breaking in that if followed, this approach has the potential to set the system on an entirely new path that differs from past norms.

### 3.6 Approach 6: systems first

The System First approach uses systems thinking to develop FBDGs and shifts the focus from ‘food-based’ to ‘food system-based’ guidelines. The FAO has recently proposed food system-based dietary guidelines (FSBDGs) (74). The insertion of ‘systems’ is significant because it recognises the need for using a ‘multi-criteria’ approach to food (75) and the need for using systems thinking then developing FSBDGs. FSBDGs should contribute towards achieving the Sustainable Development Goals and provide guidance on how to embed sustainability (including environmental sustainability) throughout the process of developing guidelines (personal communication A.I. Ramos, FAO, February 2022). Specifically, FSBDGs are described by FAO as:

… context-specific multilevel recommendations that enable governments to outline what constitutes a healthy diet from sustainable food systems, align food-related policies and programmes and support the population to adopt healthier and more sustainable dietary patterns and practices that favour, among other outcomes, environmental sustainability and socio-economic equity. Their effectiveness resides in that they are developed through an evidence-informed, multidisciplinary and multisectoral engagement process and with a food system approach. They result in a package of outputs and resources that can be adopted and used for guiding food system transformation towards better diet-related practices and, subsequently, better health, better nutrition, and more sustainable and equitable food systems… (74).

Currently, no country has adopted FSBDGs, mostly due to their infancy and that the methodology is yet to be released. FAO plans to release a detailed methodology in 2024, followed by a series of webinars to facilitate their uptake. However, the process for adopting FSBDGs will follow six, iterative stages:

1. Design and plan the national process
2. Analyse the situation and review the evidence
3. Develop recommended dietary patterns and formulate the technical recommendations
4. Develop the national implementation strategy
5. Design communication and capacity development actions; and
6. Implement, monitor and evaluate (74).

There is currently no detailed, stepwise framework that systematically incorporates environmental sustainability as a primary consideration. Wood et al. (19) piloted such a framework for Sweden with food-system elements by performing a stepwise simulation to iteratively adjust a healthy diet in order to improve environmental outcomes. However, this framework is yet to be implemented.

One strength of the FSBDGs put forth by the FAO is the focus on implementation, following a theory of change including intermediate and long-term outcomes (74). The theory of change draws from the conceptual framework of sustainable food systems for better nutrition, which was established by the High-Level Panel of Experts on Food Security and Nutrition (76). It includes how FBDGs as an intervention can lead to the fulfilment of the desired goals (SDGs and/or PBs).

#### 3.6.1 Level of impact

The Systems First approach can be considered as path-breaking in that it has the potential to be a disruptive innovation by considering health and the environment equally and creating major changes by setting the food system on an entirely new pathway that differs from past norms. This approach can help prioritise competing demands and if well applied, could also support the 12 dimensions of the social foundation in the Doughnut-model of Raworth (77). These 12 dimensions are derived from internationally agreed minimum social standards, as identified by the world’s governments in the Sustainable Development Goals in 2015 (37). To make a real systemic transformation happen, the establishment of not just an enabling but a normative environment, guiding the technological innovation process is needed (29).

## 4 Discussion and policy implications

**Figure 2:**
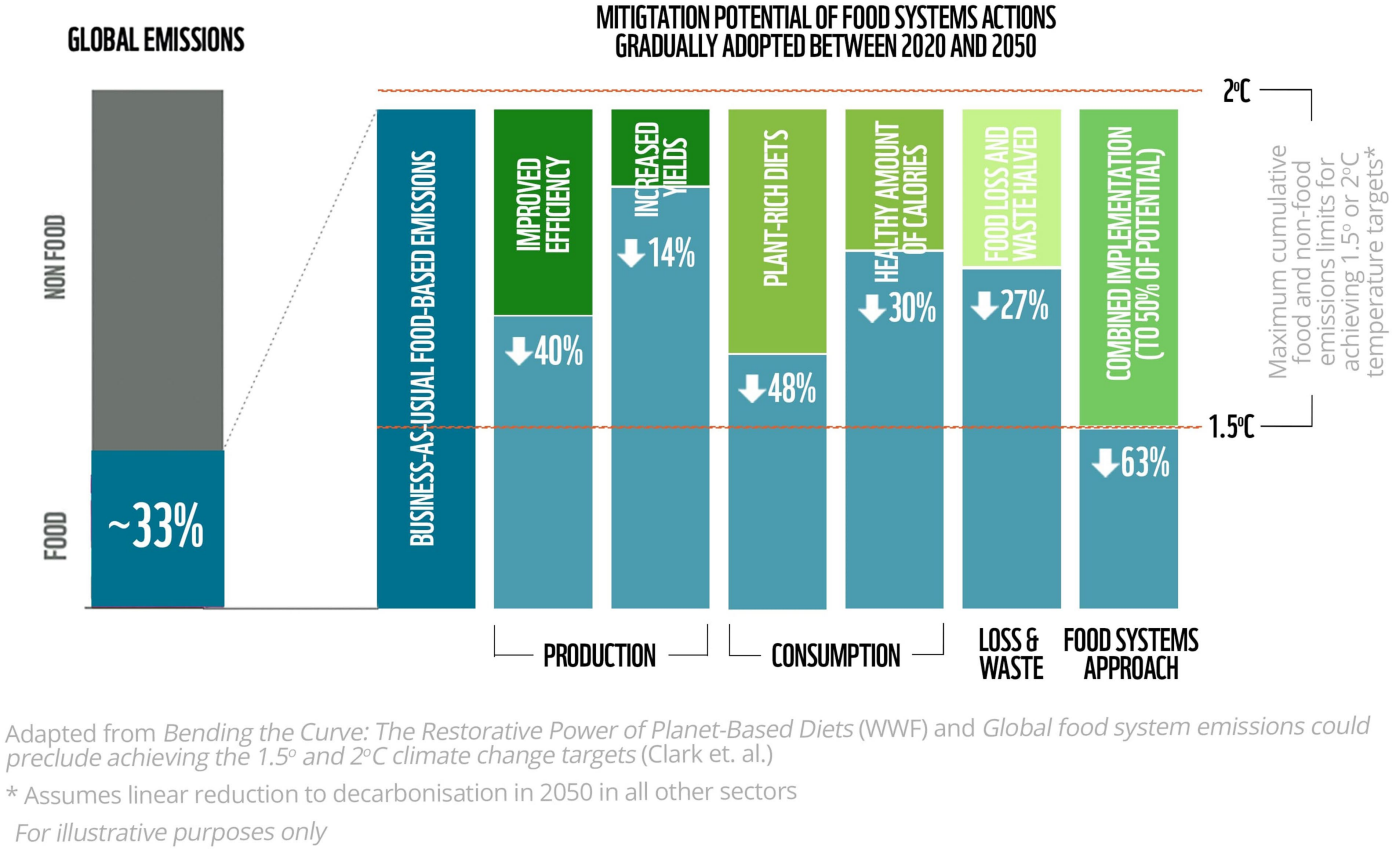
Food systems account for roughly a third of global GHG emissions and if ignored (i.e., Business-As-Usual) will use the remaining carbon budget to stay within 2°C of warming. However, if a food system approach is used, addressing production, consumption and reductions in food loss and waste, food based GHG emissions can be reduced to be in line limiting warming to 1.5°C. Of all actions, dietary shifts have the single largest impact for emissions reductions (WWF; (40)).

The paper has outlined six different approaches for incorporating environmental sustainability into FBDGs and discussed the need to raise the ambition of current guidelines so that future FBDGs are more disruptive. In a world in which climate change, biodiversity loss, food security and diet-related illnesses are all of major concern, dietary shifts towards ‘planet-based diets’ (78) have been shown to be one of the most effective actions for addressing the multiple converging environmental and health crises we are experiencing today. To help achieve these dietary shifts, developing ambitious national FBDGs may provide the single greatest opportunity for policymakers to develop coherent food and agricultural priorities across all parts of the food system to alleviate all forms of malnutrition while reducing the environmental impact of food systems (Figure 2). Despite this, most FBDGs are not ambitious enough to achieve both health and environmental goals (15). In addition, when revisions to FBDGs do happen, countries tend to adopt incremental approaches when either diversifying or path-breaking approaches are needed to solve the urgent problems at hand.

However, there are a few countries that have taken significant steps in fully integrating environmental sustainability into their FBDGs and important lessons can be learned from those countries (e.g., Netherlands, Denmark). But not all countries have either the expertise, capacity or financial resources or commitment of, for example, the Nordics, Canada, Belgium, and the Netherlands to carry out such a detailed and resource-intense process as described especially in Approaches 3, 4 and 5. Therefore, the choice of approach chosen by a country may depend not only on their level of ambition but also on the experience, budget, data, and time available as well as opportunities and avenues for policy influence. For those countries with limited capacity and or resources, support from FAO is necessary, as is the exchange of experience between countries. The WHO has also developed several work streams to support countries in their requests for greater clarity over how to change dietary patterns (24).

### 4.1 Why is adoption and implementation of path-breaking FBDGs so difficult?

Our paper proposes that there have been several different approaches to how countries have integrated environmental sustainability into FBDGs, in other words, how to tackle the concept of sustainable diets in order to recommend healthy and sustainable diets in FBDGs. This diversity of approaches and lack of a unified coherent position on sustainable diets, despite the recommendation from the FAO, WHO, and scientific community, indicates that policy-makers may still feel sustainable diets are a ‘tricky’ concept to take on politically or it may also be due to a lack of good data and experience on how to develop guidelines that fully integrate environmental sustainability.

Why is this the case? To date, few governments have wanted to engage with the multi-criteria messages (75) about the science-based synergies between health and sustainability. Their policy concerns have been mostly about cost – keeping rising populations fed with enough, safe and healthy foods. In addition, food politics has also been changed by consumerist culture which centres on individual consumer choice. Civil society organisations (with some exceptions) have been indifferent. Taken together, this has created a policy situation that can be summarised as ‘leave it to consumers’ plus ‘leave it to market dynamics.’

The food industry (i.e., Big Food) has also met the sustainable diet challenge with a mixture of commercial reluctance (‘it’s too hard’) and hostility (‘it will undermine processed food markets’). Béné argued that concentration of market power in the hands of the Big Food transnational corporations, together with ideology, policy incoherence, national interests and culturally-embedded aspirations create irreconcilable trade-offs and tensions and prevent the system from aligning toward a more sustainable trajectory (29).

However, the problem for integrating environmental sustainability into FBDGs is a strategy more than just policy (focusing on how to get change, not exclusively on the end goal) (74). In this paper, the strong evidence that food consumption has an impact on both health and the environment has been our starting point. Responses to that evidence, however, have been (a) slow to emerge, (b) seemingly unable to prevent impacts, (c) received much opposition from lobbies, and (d) limited to national scale. The examples given in Approaches 1, 2, and 3 highlight that barriers exist to fully integrate the concept of environmental sustainability into FBDGs.

The challenge of integration of environmental sustainability into FBDGs, which would mean acceptance of healthy and sustainable diets as a concept, is not just a challenge to society (e.g., tackling climate change) or the food system (e.g., building resilience into the food system), it is also a challenge to governance: companies can be threatened, special interests can be threatened, the economic doctrine that consumer choice drives the food economy can be threatened.

Our paper provides an overview of the approaches that have been used to integrate environmental sustainability (i.e., sustainable diets) into FBDGs. Approaches 1 is the safest approach and does very little to disrupt the status quo or challenge governance at large. Approaches 2, 3 and 4 take important steps in the direction of integrating environmental sustainability into FBDGS but still lead to mainly smaller changes or sustaining the status quo. However, only approaches 5 and 6 can be considered path-breaking in that they are meant to set the system on an entirely new pathway that diverges from past norms.

It is no wonder why so few countries have used these approaches to date, and the countries that have used them (e.g., Netherlands, Denmark) still face challenges with universal adoption by the general public. Yet, only these approaches can help meet global health and environmental sustainability goals, as long as the environmental constraints used in the modelling are ambitious enough to achieve the SDGs, Paris Agreement, and the Global Biodiversity Framework. Anything short of ambitious path-breaking approaches will preclude our chances of tackling the multiple converging crises we are facing in climate change, biodiversity loss and the triple burden of disease (79).

There are several reasons why implementing path-breaking FBDGs is so difficult. FBDGs should be seen as complementary to strategic, comprehensive, and culturally appropriate dietary and health promoting interventions, and not only as a tool for providing information on nutrition and the environment. Other barriers to implementation include lack of funding for FBDGs compared to commercial advertising, lack of political support for developing coherent policy around FBDGs or because commerce finds health demands difficult enough, without addressing environmental demands as well.

Another major challenge with integrating environmental sustainability into FBDGs is that instead of culture and economy defining a good diet as ‘what we like’ and ‘what is available and affordable’, a healthy and sustainable diet is judged by multiple criteria and policymakers can find multi-criteria thinking difficult (75). Without knowing, proponents of healthy and sustainable diets have championed multi-criteria analysis and are asking governments, the public and industry to reshape how they judge food – and to do this very fast as the climate and nature crises require.

But adopting multi-criteria thinking is essential when prioritising competing demands in a complex world. In truth, we have always applied multiple criteria to judge food. Table 3 sets out some broad distinctions between types of multi-criteria guidelines for judging food. One can identify ‘Old multi-criteria guidelines’ that judged good food by, for example: size, shape, colour, smell, taste, cost, availability, familiarity, experience, culture, and religious rules. With the nutrition transition, as economies became more affluent, the ‘New multi-criteria guidelines’ emerged: massive choice, newness, excitement, modernity, packaging, marketing, branding, speed, and time.

**Table 3:** Types of multi-criteria guidelines for judging food.

Types of guidelines	Era	Food tends to be judged by criteria such as …	The criteria are shaped by …
Old	Much of human history	Size, shape, colour, smell, taste, cost, availability, familiarity, experience, culture, religious rules	What is available; cultural and religious rules; regional food availability
New	20th century	Growth of choice, newness, excitement, modernity, packaging, marketing & branding, speed, time	New industrial and production techniques and foods; growth of food trade
New 3.0 or Emerging	21st century	Impacts on climate, water use, biodiversity, public health, fairness, morality, simplicity, ‘hidden’ waste, animal welfare, circularity	Rising awareness of food’s sustainability crisis and its long-term not just short- term effects

Adding environmental sustainability into FBDGs is thus a battleground about old and new guidelines onto which we want to add a new layer: ‘New 3.0 or emerging multi-criteria guidelines’ which include: climate change, biosphere integrity, freshwater consumption, novel entities, impacts on natural resources, fairness, animal welfare, and circularity. These criteria are increasingly used to define what is meant by a good diet or food or food system. In addition, Fanzo et al. (80) proposed an audit of food systems change based on criteria including resilience and sustainability, livelihoods, poverty, and equity. What is needed is not just more data or more evidence, but different types of data and evidence, in particular at the interface between science, society and policy in relation to food systems (29).

Opponents of healthy and sustainable diets may claim that the ‘New 3.0 multi-criteria guidelines’ take the fun (freedom of choice) out of consumer choice. We disagree. There will continue to be ample choice but some constraints on food choices are needed to ensure a long-term thriving future for humanity. We therefore consider some possible recommendations for policymakers faced with negotiating this new complexity.

### 4.2 Recommendations for policy makers

The analysis presented here implies some change in direction for many food-related policy actors. Today, the ambition to feed all people healthily is not enough. We also must also consider how to produce this food sustainably. Understandably, this new but urgent path for the 21st century can create divisions among policy makers who may find addressing sustainable diets too difficult. This new path can also surface social and economic divisions. Low and middle income countries (LMIC) may accuse High Income Countries (HICs) of advising them (again) to do what they have not done themselves while all countries suffer the health and environmental consequences of the rapid spread of unhealthy and unsustainable foods. In addition, LMICs must also urgently address the burden of hunger and undernutrition, while many HICs must address issues of overconsumption. Given the very different social and economic conditions faced by countries, our recommendations below are carefully phrased to allow for those very different circumstances.

Above all, the healthy and sustainable diet challenge requires collaborative action. No-one can claim their national situation is impregnably sound or immune to the risks ahead. Nor is there a single policy lever that can even dent the problem now facing humanity. Table 4 presents six key points of entry into this global policy challenge.

**Table 4:** Six recommendations for policymakers faced with negotiating this new complexity of ‘New 3.0 multi-criteria guidelines’.

1	Understand that going from Health First to Systems First FBDGs is a process that may challenge existing governance, norms, institutions, and ways of looking at food.
2	Strive for Systems First FBDGs while also considering the advantages and disadvantages (Table 2) if other approaches are adopted.
3	If a Systems First approach is used, learn from other countries who have used either this or a Combined Strategy approach to understand potential barriers to success.
4	Consider cooperation among countries by creating regional guidelines like the Nordic Nutrition Recommendations, which can be a resource-saving solution.
5	When developing or updating FBDGs, reach out to the FAO and WHO and learn more about their six-stage methodology for developing food systems based dietary guidelines (FSBDGs).
6	Invest in research into better understanding the barriers to adopting FBDGs and how these barriers can be lowered to ensure they are widely adopted.

Firstly, societies must see the case for change. This requires a wide range of policy actors in government (at all levels), civil society, food business and science to try to speak with more unified voices on that case for change. No-one will be unaffected. Societies must either begin to address the challenge or else change is likely to be forced on them later by events in worsening crises.

Secondly, public health and diet-related professions must accept, however reluctantly, that the old era of FBDGs is coming to a close. We can no longer argue that food must only be viewed through the lens of nutrients. Food’s embedded environmental impact might matter as much, and sometimes more.

Thirdly, coalitions of interest should be created to agree on their Combined Strategies – how they are going to aid and improve the process of change to healthy and sustainable diets. This will manifest differently in LMICs than in HICs.

Fourthly, even countries that produce almost all of their food now live in a world where actions in one can affect others. Food illustrates how the world is connected, if not in actual transfer of food, then of tastes, styles, aspirations and knowledge. Professional bodies with solid international links can help that global learning.

Fifthly, the FAO and WHO remain the ‘peak’ relevant bodies through which exchange can happen. UN bodies are sometimes criticised for being an arena where vested national interests emerge, however food is and must be a focus for cooperation.

Sixthly, with the emergence of multi-criteria approaches to food systems and diets, the need for solid science and evidence becomes even more important than usual. Policy analysts like to aim for ‘evidence-based policy’ when, as in this case, what is needed is more evidence that illuminates policy options, or ‘policy-based evidence’.

### 4.3 Future research directions

This paper outlines six approaches that have been used in the development of FBDGs. Past research has focused on the healthiness and sustainability of FBDGs (15, 16) and also on the methodologies used in FBDGs. Robust methodologies, including the recently developed FSBDGs (74) now exist that can guide countries in the process of developing ambitious FBDGs that incorporate both health and environmental sustainability. However, very little research has been devoted to the implementation of FBDGs and the barriers that may enhance or hinder their adoption.

Although a common methodology can be helpful to guide the development of FBDGS, there is no one-size-fits-all solution that can drive the successful implementation of FBDGs in all countries around the world. Different regions and nations face a range of diverse opportunities and challenges shaped by local ecology, culture, histories and levels of development. Given this, implementation of FBDGs needs to be place-based and future research should focus on understanding the enabling conditions in each country that would support successful implementation.

Understanding these details is very important. But it is equally as important to not become mired in or overwhelmed by the endless complexity that exists from country to country and miss opportunities to learn from or replicate effective solutions. Typologies can be helpful for reducing complexity to a level where we can work with it and learn from it, rather than being overwhelmed by it. For example, WWF developed a global food systems typology that integrates both health and environmental variables and identified key transformation levers for each food system type (81). In addition, the World Economic Forum adopted a typology from Marshall et al. (82) to help inform country level roadmaps for food, nature, and health transitions. And finally, FAO plans to release a detailed methodology for FSBDGs in 2024 (74). Future research can build on this promising line of inquiry to help reduce the complexity of successful implementation of FBDGs by identifying repeatable country models that are based on local conditions.

## 5 Conclusion

The paper has outlined six different approaches for incorporating environmental sustainability into FBDGs and the need to raise the ambition of current guidelines. As Mason and Lang comment, ‘SDGs cannot be met unless the food system changes. The food system cannot change unless consumers change, and the consumer change needs to be shaped by clear evidence-informed guidelines, hence the case for Sustainable Dietary Guidelines’ (i.e., FSBDGs) (35). The government’s role within this web of actors is to lead, facilitate updates and publish science-based FBDGs that incorporate environmental and social sustainability.

There is a growing consensus for the need of a food systems approach to address the complexity of food system transformation. There has been a disconnect in the past amongst researchers, policymakers, politicians, and practitioners, and some reluctance of governments to apply multi-criteria thinking has followed (75). Today, there is growing scientific consensus that foods that are good for human health are also good for the environment. There is also a growing recognition that food system change is inevitable and desirable, shared by nutritionists, environmentalists, other scientists concerned about food’s impacts and even sections of food industries resistant in the past. We see this as a positive opportunity to collaborate on FBDGs more appropriate for the 21st century. Discussions over guidelines and food systems already acknowledge the sociocultural dimensions of the sustainability agenda, where food constitutes a major cultural transmission link. We see the sustainable diet challenge as contributing to this new consensus. It is important not only to develop healthy and sustainable FBDGs, but also to implement them, and to help consumers adopt the diets being recommended. Coherence and consensus about new healthy and sustainable FBDGs could help engage with civil society and the media and reinforce the importance of sustainable food systems in the political agenda. Teamwork between scientists, policymakers, politicians, and practitioners is essential (83), especially further research into better understanding the barriers to adopting FBDGs and how these barriers can be lowered. Importantly, given the very different social and economic conditions faced by countries, the development and implementation of FBDGs must be place based and consider local realities. Given sustainability and systemic pressures on food supply and consumption, we expect that further improvement of 21st century FBDGs, refinement of methodologies and lowering of barriers for implementation will be critical, not least if crises accelerate. The case for healthy and sustainable diets is unlikely to disappear.

## Data availability statement

The original contributions presented in the study are included in the article/supplementary material, further inquiries can be directed to the corresponding author.

## Author contributions

CD developed the approaches and design of the study with input from all authors and performed the literature research and wrote the first draft of the manuscript. SH, HM, LN, MS-S, and ET wrote sections of the manuscript related to their country. All authors contributed to manuscript revision, read, and approved the submitted version.
